# Meta-Analysis of Randomized Controlled Trials Involving Anterior Shoulder Instability

**DOI:** 10.2174/1874325001812010411

**Published:** 2018-10-25

**Authors:** Kavin Khatri, Hobinder Arora, Sanjay Chaudhary, Darsh Goyal

**Affiliations:** 1 Department of orthopaedics, GGS Medical College, Faridkot, India; 2 Department of community medicine, GGS Medical College, Faridkot, India; 3 Department of orthopaedics, Sport injury center, Safdarjung Hospital, New Delhi, India

**Keywords:** Arthroscopic, Shoulder, Bankart, Anterior instability

## Abstract

**Background:**

Arthroscopic repair is gaining popularity over open repair for the treatment of bankart lesions. The study aims to evaluate the outcome of arthroscopic repair with open repair in randomised controlled trials conducted comparing the two techniques.

**Methods:**

We searched the Cochrane library, PubMed and EMBASE up to December 2017 for clinical trials comparing the outcomes of arthroscopic bankart repair with open bankart repair. We used fixed or random effects model depending upon heterogenicity. Dichotomous variables were presented as Risk Ratios (RRs) with 95% Confidence Intervals (CIs), and continuous data were measured as measured differences with 95% CIs.

**Result:**

Five studies were included, with sample size ranging from 42 to 196. Fixed effect analysis showed that the shoulder was more stable in open repair (RR=0.897, 95% CI: 0.821 to 0.980, P= 0.94) but the loss of external rotation at shoulder joint was also higher in those had open repair (RR=0.325, SMD=-0.411, 95% CI: -1.229 to 0.407). The functional outcome assessed by Rowe score was better in open repair (P=0.325). The operative time was lesser in arthroscopic repair but was not statistically significant (P=0.085).

**Conclusion:**

Our meta-analysis showed that the use of arthroscopic repair though offers better shoulder movement but the open repair is superior in terms of shoulder stability.

## 1 INTRODUCTION

Treatment of choice for Bankart lesion with anterior shoulder instability is mired with controversy even with the introduction of newer and advanced surgical techniques. Bankart had advocated the anatomical repair of the capsulolabral complex [1, 2]. Traditional open bankart repair has been accepted as standard in treating this condition by many surgeons [2]. Open bankart repair has shown to improve shoulder joint stability with low recurrence and failure rate [3, 4]. In last decade, the arthroscopic repair has gained popularity in the treatment of this injury. Advantages like shorter hospital stay, early rehabilitation, lower complication rate and lesser soft tissue damage in comparison to open techniques has worked in its favour [5, 6]. However, the higher incidence of redislocation has been noted with the arthroscopic techniques [6, 7]. 

Several studies have compared the benefit of either technique over the other but the published data lack powerful evidence [8-11]. Randomised trials have been conducted in recent years to establish the superiority of one technique over another [4, 6]. The high recurrence rate which was earlier reported with the arthroscopic techniques has now shown to decrease over the time [9]. The aim of the current study was to conduct a meta-analysis of randomised control trails comparing the results of traditional open bankart repair and arthroscopic bankart repair in traumatic anterior shoulder instability. The study shall obtain the best evidence in managing the cases of anterior shoulder instability.

## 2 MATERIAL AND METHODS

### 2.1 Data Sources

The review was conducted in accordance with guidelines prescribed in Cochrane handbook for systemic review and meta-analysis of interventions. We searched Medline PubMed, Embase and Cochrane library for published data using the following medical subject heading terms (mesh), keywords and their combinations: open bankart repair, anterior shoulder instability, Bankart repair, suture anchors, arthroscopic Bankart repair, Bankart lesion, dislocation, and subluxation. The search was limited to studies in humans published in English literature. The bibliographies of the retrieved articles and other relevant publications, including reviews and meta-analysis, were cross-referenced for additional information and articles. The following Web sites were searched to identify the unpublished data and ongoing studies: Current Controlled Trials (www.controlled-trials.com); Trials Central (www.trialscentral.org/ClinicalTrials.aspx) and Center Watch (www.centerwatch.com). The bone and joint journal (http://www.bjj.boneandjoint.org.uk), the journal of bone and joint surgery (www.ejbjs.org) and the American Academy of Orthopaedic Surgeons (www.aaos.org) were searched manually.

### 2.2 Eligibility Criteria:

All randomized control clinical trials published in English literature comparing the open bankart repair with arthroscopic bankart repair were included in the study. We excluded cadaveric, biomechanical studies and studies that provided insufficient information on population characteristics, surgical procedure, or outcome.

### 2.3 Study Identification and Data Extraction

Two authors (KK and HA) independently screened all the titles and abstracts identified from the search strategy. The articles were then reviewed again by other authors (SC, DG) against the predefined criteria. The authors (KK and HA) then independently reviewed each shortlisted paper. Each reviewer extracted data on a predefined database. The two databases were then compared. Disagreement between the authors was resolved by discussion. The methodological quality of each Randomized Controlled Trial (RCT) was assessed using Physiotherapy Evidence Database (PEDro) scale [12].

### 2.4 Outcome Measures

Four parameters namely shoulder stability, loss of external rotation, functional outcome and surgical time were assessed. The shoulder was considered stable if there was no episode of dislocation after surgical intervention. External rotation is the mostly affected after bankart repair. The assessment of the external rotation was carried out with shoulder in abduction. the functional outcome was measured using Rowe score [13]. Rowe score consists of a total of 100 points divided into three spheres: (a) stability, which included a total 50 points; (b) mobility, which corresponds to 20 points; (c) function, which included 30 points. The score is considered excellent between 90 to 100 points, good between 89 and 75 points, fair ranging between 74 and 51 points and poor below 50 points. The operative time was calculated from the time of skin incision to closure.

### 2.5 Statistical Analysis

The statistical analysis was carried out by the single author (KK) using Review Manager 5.3 (Nordic Cochrane Centre, Cochrane Collaboration 2009, Copenhagen, Denmark). The Chi square test was performed to evaluate heterogeneity of the data and was determined to be significant at χ2>50%. A fixed-effect model was used when the heterogeneity was not significant and random-effects model was used if the significant heterogeneity was noticed. Funnel plot was used to evaluate the publication bias. A probability of p<0.05 was determined as significant, and 95% confidence intervals (CIs) were also calculated.

## 3 RESULTS

2361 abstracts were obtained based upon the search strategy. We identified 68 articles that had comparison between arthroscopic bankart repair and open bankart repair. They were scrutinised based upon the inclusion criteria and five studies were included in the final assessment [13-17]. Only level one studies (randomised controlled trails) were included in the meta-analysis. The methodological quality of studies was assessed using Physiotherapy Evidence Database (PEDro) scale and has been tabulated in Table **I**.

The number of patients included in these randomized controlled trials ranged from 42 to 196. A total of 415 patients were enrolled in the randomized controlled trials, including 331 men (79.75%) and 84 women (20.24%). The mean age ranged from 24.5 to 30.8 years. The details are shown in Table **2**.

### 3.1 Statistical Analysis

**Fig. (1):**
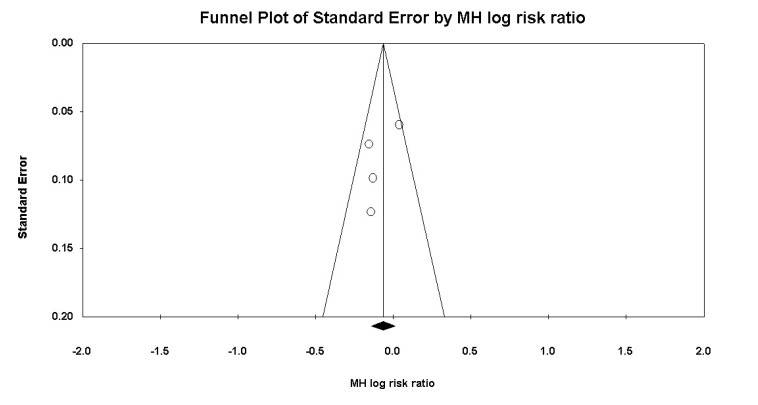
Funnel plot of standard error by MH log risk ratio.

The statistical analyses were conducted using Review Manager 5.3. The dichotomous data were analysed by MH Risk Ratios (RR), and continuous data by Weighted Mean Difference (WMD), both with 95% Confidence Intervals (CIs). Chi-squared test was performed to evaluate heterogeneity of studies. Funnel plot was used to evaluate the publication bias (Fig. **1**).

### 3.2 Shoulder Stability:

The stability at shoulder joint was assessed using fixed-effects model Fig. (**2**). A statistically significant difference was observed between the OBR and the ABR group in respect to shoulder stability (*P*=0.016, RR=0.897, 95% CI: 0.821 to 0.980). The OBR group had better shoulder stability in comparison to ABR.

### 3.3 Loss of External Rotation at Shoulder

Loss of external rotation at shoulder joint is seen after bankart repair. Using random effect model, the result of analysis revealed a better range of motion in patients managed with ABR, compared to those with OBR (*P*=0.325, SMD=–0.411, 95% CI: –1.229 to 0.407) but the results were not statistically significant Fig. (**3**). The external rotation is observed to decrease more after OBR.

### 3.3 Functional Outcome

Various parameters like Rowe score [18], Constant score [19], University of California, Los Angeles(ULCA) shoulder score [20], Western Ontario Instability Index (WOSI) score [21] were noted in studies to assess the functional outcome. However, due to lack of universally acceptable functional outcome assessment measures in shoulder, the comparison between the studies was difficult. The meta-analysis using fixed effects model Fig. (**4**) revealed a statistically significant difference between the two treatment strategies (*P*=0.035). The functional outcome is better in open bankart repair. The analysis result, however, needs to be interpreted cautiously due to lesser number of studies.

### 3.4 Surgical Time

Intraoperative surgical time included time of incision to complete wound closure. There was no statistically significant difference between the two groups (P=0.085, SMD=–2.036, 95% CI: –4.353 to 0.280) Fig. (**5**). However, arthroscopic procedure was performed in a shorter time as compared to open bankart repair.

## 4 DISCUSSIONS

The present study has shown that the Arthroscopic Bankart Repair (ABR) offers better shoulder movement but open repair is superior in terms of shoulder stability. Open Bankart Repair (OBR) is considered as gold standard in the management of a case of shoulder instability. The technique described by Rowe **et al*.* [2] has stood the test of time. OBR involves the dissection of subscapularis and humeral capsule to identify the detachment portion of the labrum which then can be attached using various techniques.

ABR aims to achieve all the benefits of OBR without the bad effects like pain, blood loss and longer time for rehabilitation. The initial results of ABR were discouraging with higher rate of redislocation [22, 23]. The principal reasons for failure were inability to identify associated bony lesions, significant learning curve, failure to address capsular laxity [24, 25].

In the recent years, there has been significant improvement in the results obtained with ABR and hence debate is again shifting to consider it as equivalent to OBR. In the past, few meta- analysis has been conducted comparing the results between two techniques however none has included randomised controlled trials exclusively to formulate the opinion [4, 22].

In the meta-analysis, it was seen that the patients who had ABR were better in terms of external rotation at shoulder in comparison to patients who had undergone OBR [22].

The strength of our meta- analysis is that we had included only randomised control trails for analysis. With our strict inclusion criteria, only five studies with 415 subjects were considered. Combining and evaluating the results between 207 cases of ABR and 208 cases of OBR, we found lower rates of redislocation in ABR and the results were statistically significant (P=0.016).

The limitations of the study were non-homogenous populations and different studies have stressed on different outcome measurement assessment scales thus making comparison difficult variability in outcome assessment measurement. Factors like level of individual activity, degree of instability (dislocation *vs* instability) were not reported in each study. The sample size was relatively small except in one study by Mohatadi *et al.* [17] The level of expertise also varies considerably in various surgeons opting for either ABR or OBR.

## CONCLUSION

The study observed lower range of motion but better shoulder stability in OBR group. However, the difference was not statistically significant in terms of movement at shoulder joint but the stability was certainly better than arthroscopic group. Larger randomised controlled trials are needed to draw strong conclusions regarding the superiority of one technique over the other.

## Figures and Tables

**Fig. (2):**
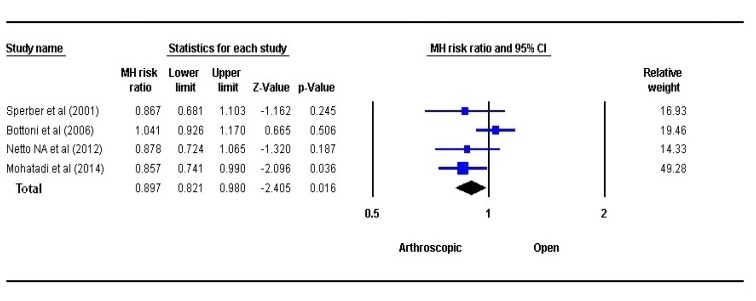
The stability at shoulder joint was assessed using fixed-effects model.

**Fig. (3):**
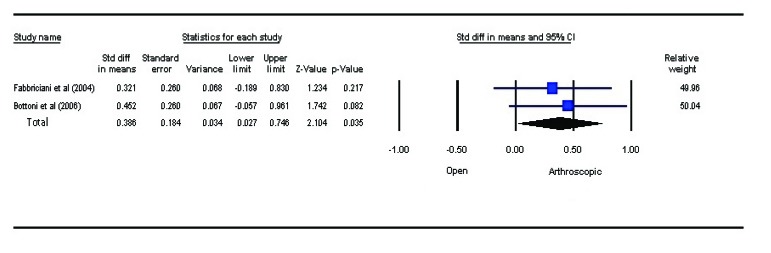
The external rotation is observed to decrease more after OBR.

**Fig. (4):**
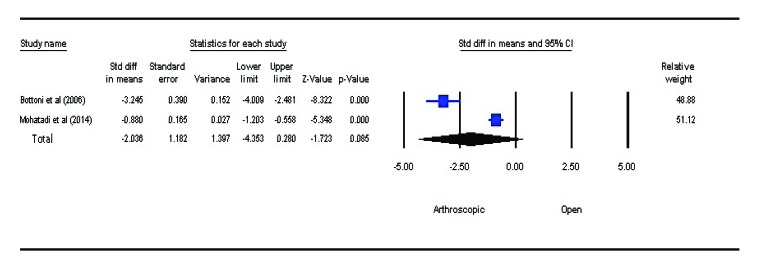
The meta-analysis using fixed effects model.

**Fig. (5):**
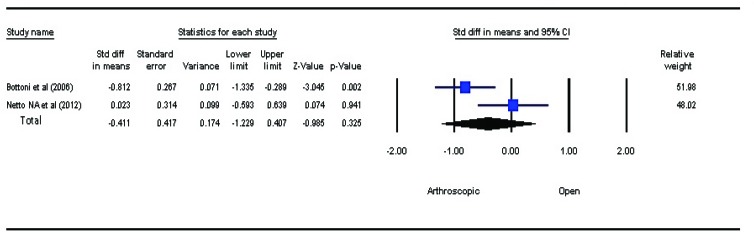
Arthroscopic procedure.

**Table I:** Basic characteristics of the studies included in meta-analysis.

Study	No. of Patients		Sex (male/female)		Mean Age (in years)		Mean Delay in Surgery (months)		Mean Follow up Period (months)	
	Ac	Open	Ac	Open	Ac	Open	Ac	Open	Ac	Open
Sperber *et al* (2001) [13]	30	26	40	16	25	27.5	57.6	42	24	24
Fabbriciani *et al* (2004) [14]	30	30	50	10	24.5	26.8	25.3	20.2	24	24
Bottoni *et al* (2006) [15]	32	29	60	1	25.2	25.1	35.1	41.6	28.5	30
Netto NA *et al* (2012) [16]	17	25	16	21	27.5	30.8	45.7	62.9	37.5	37.5
Mohatadi *et al* (2014) [17]	98	98	160	36	27.2	27.8	54	75	24	24

Ac: arthroscopic technique; open: open technique

**Table 2:** Assessment of studies on Physiotherapy Evidence Database (PEDro) critical appraisal scale.

Criteria	Sperber *et al* (2001)^13^	Fabbriciani *et al* (2004)^14^	Bottoni *et al* (2006)^15^	Netto NA *et al* (2012)^16^	Mohatadi *et al* (2014)^17^
Were eligibility criteria clearly specified	yes	yes	yes	yes	yes
Random allocation of subjects	yes	yes	yes	yes	yes
Concealment of allocation	Yes	yes	yes	yes	yes
Similarity in groups regarding baseline characteristics	yes	yes	yes	yes	yes
Blinding of all subjects	no	no	no	no	no
Blinding of all clinicians	no	no	no	no	yes
Blinding of all assessors	no	no	yes	no	no
Adequate follow up	yes	yes	yes	yes	yes
Intention to treat analysis	no	no	no	no	yes
Reporting of statistical results between groups	yes	yes	yes	yes	yes
Provision of point measure and measures of variability	yes	yes	yes	yes	yes
Total score	7	8	9	7	9

## References

[1] Bankart A.S.B. (1938). The pathology and treatment of recurrent dislocation of the shoulder-joint.. Br J. Surg..

[2] Rowe C.R., Patel D., Southmayd W.W. (1978). The Bankart procedure: A long-term end-result study.. J. Bone Joint Surg. Am..

[3] Walch G., Boileau P., Levigne C., Mandrino A., Neyret P., Donell S. (1995). Arthroscopic stabilization for recurrent anterior shoulder dislocation: Results of 59 cases.. Arthroscopy.

[4] Freedman K.B., Smith A.P., Romeo A.A., Cole B.J., Bach B.R. (2004). Open Bankart repair *versus* arthroscopic repair with transglenoid sutures or bioabsorbable tacks for Recurrent Anterior instability of the shoulder: A meta-analysis.. Am. J. Sports Med..

[5] Wang C., Ghalambor N., Zarins B., Warner J.J. (2005). Arthroscopic *versus* open Bankart repair: Analysis of patient subjective outcome and cost.. Arthroscopy.

[6] Green M.R., Christensen K.P. (1993). Arthroscopic *versus* open Bankart procedures: A comparison of early morbidity and complications.. Arthroscopy.

[7] Hobby J., Griffin D., Dunbar M., Boileau P. (2007). Is arthroscopic surgery for stabilisation of chronic shoulder instability as effective as open surgery? A systematic review and meta-analysis of 62 studies including 3044 arthroscopic operations.. J. Bone Joint Surg. Br..

[8] Uhorchak J.M., Arciero R.A., Huggard D., Taylor D.C. (2000). Recurrent shoulder instability after open reconstruction in athletes involved in collision and contact sports.. Am. J. Sports Med..

[9] el Akad A.M., Winge S., Molinari M., Eriksson E. (1993). Arthroscopic bankart procedures for anterior shoulder instability. A review of the literature.. Knee Surg. Sports Traumatol. Arthrosc..

[10] Geiger D.F., Hurley J.A., Tovey J.A., Rao J.P. (1997). Results of arthroscopic versus open Bankart suture repair.. Clin. Orthop. Relat. Res..

[11] Speer K.P., Deng X., Borrero S., Torzilli P.A., Altchek D.A., Warren R.F. (1994). Biomechanical evaluation of a simulated Bankart lesion.. J. Bone Joint Surg. Am..

[12] Maher C.G., Sherrington C., Herbert R.D., Moseley A.M., Elkins M. (2003). Reliability of the PEDro scale for rating quality of randomized controlled trials.. Phys. Ther..

[13] Sperber A., Hamberg P., Karlsson J., Swärd L., Wredmark T. (2001). Comparison of an arthroscopic and an open procedure for posttraumatic instability of the shoulder: A prospective, randomized multicenter study.. J. Shoulder Elbow Surg..

[14] Fabbriciani C., Milano G., Demontis A., Fadda S., Ziranu F., Mulas P.D. (2004). Arthroscopic *versus* open treatment of bankart lesion of the shoulder: A prospective randomized study.. Arthroscopy.

[15] Bottoni C.R., Smith E.L., Berkowitz M.J., Towle R.B., Moore J.H. (2006). Arthroscopic versus open shoulder stabilization for recurrent anterior instability: A prospective randomized clinical trial.. Am. J. Sports Med..

[16] Archetti Netto N., Tamaoki M.J., Lenza M., dos Santos J.B., Matsumoto M.H., Faloppa F., Belloti J.C. (2012). Treatment of Bankart lesions in traumatic anterior instability of the shoulder: A randomized controlled trial comparing arthroscopy and open techniques.. Arthroscopy.

[17] Mohtadi N.G., Chan D.S., Hollinshead R.M., Boorman R.S., Hiemstra L.A., Lo I.K., Hannaford H.N., Fredine J., Sasyniuk T.M., Paolucci E.O. (2014). A randomized clinical trial comparing open and arthroscopic stabilization for recurrent traumatic anterior shoulder instability: Two-year follow-up with disease-specific quality-of-life outcomes.. J. Bone Joint Surg. Am..

[18] Rowe C.R., Zarins B. (1981). Recurrent transient subluxation of the shoulder.. J. Bone Joint Surg. Am..

[19] Constant C.R., Murley A.H. (1987). A clinical method of functional assessment of the shoulder.. Clin. Orthop. Relat. Res..

[20] Amstutz H.C., Sew Hoy A.L., Clarke I.C. (1981). UCLA anatomic total shoulder arthroplasty.. Clin. Orthop. Relat. Res..

[21] Kirkley A., Griffin S., McLintock H., Ng L. (1998). The development and evaluation of a disease-specific quality of life measurement tool for shoulder instability. The Western Ontario Shoulder Instability Index (WOSI).. Am. J. Sports Med..

[22] Lenters T.R., Franta A.K., Wolf F.M., Leopold S.S., Matsen F.A. (2007). Arthroscopic compared with open repairs for recurrent anterior shoulder instability. A systematic review and meta-analysis of the literature.. J. Bone Joint Surg. Am..

[23] Guanche C.A., Quick D.C., Sodergren K.M., Buss D.D. (1996). Arthroscopic versus open reconstruction of the shoulder in patients with isolated Bankart lesions.. Am. J. Sports Med..

[24] Morgan C.D., Bodenstab A.B. (1987). Arthroscopic Bankart suture repair: Technique and early results.. Arthroscopy.

[25] Petrera M., Patella V., Patella S., Theodoropoulos J. (2010). A meta-analysis of open versus arthroscopic bankart repair using suture anchors.. Knee Surg. Sports Traumatol. Arthrosc..

